# Effects of *Agaricus bisporus* SMS-Garden Soil Mixture Ratios on Tomato Growth, Yield, and Quality

**DOI:** 10.3390/plants15203092

**Published:** 2026-10-09

**Authors:** Yuhan Zhao, Weijian Li, Ke Ma, Keqing Qian, Xiao Li, Haiyan Lv, Bo Zhang

**Affiliations:** 1 College of Horticulture, Jilin Agricultural University, Changchun 130118, China; 17833123288@163.com; 2 College of Mycology, Jilin Agricultural University, Changchun 130118, China; wjmogu8682@163.com (W.L.); mkkk77@163.com (K.M.); 3 Engineering Research Center of Edible and Medicinal Fungi, Chinese Ministry of Education, Jilin Agricultural University, Changchun 130118, China; 15670567112@163.com; 4 Industrial Development Institute for Plants, Animals and Fungi Integration of Biyang County, Zhumadian 463700, China; lxmogu@163.com; 5 Sanjiang Fungal Industry Collaborative Innovation Center, Changchun 130118, China; 6 Key Laboratory of Northern Edible Fungi Resource Utilization, Ministry of Agriculture and Rural Affairs, Changchun 130118, China

**Keywords:** tomato, *Agaricus bisporus*, spent mushroom substrate (SMS), growth, yield, fruit quality, sustainable agriculture

## Abstract

The present study evaluated the effects of *Agaricus bisporus* spent mushroom substrate (SMS) on growth, yield, and quality of tomato (*Solanum lycopersicum* L. cv. ‘Fenguan 888’) in a pot experiment. Ten treatments (Z1–Z10) were applied using gradient substrate replacement (SMS) ratios, relative to a control (CK) without SMS addition. The results demonstrated that SMS exerted stage-dependent growth promotion. No significant effects were observed at the seedling stage, but gradual enhancements were noted thereafter. Z6 achieved the greatest plant height increase (68.0%), while Z8 achieved the largest stem diameter increase (50.0%) at peak fruiting. Biomass accumulation was significantly enhanced, Z9 increased aboveground fresh weight by 580.7% and root length by 63.9%, while Z8 maximized aboveground dry weight (163.9%) and belowground fresh weight (312.8%). Photosynthetic pigments exhibited differential responses, Z5 maximised chlorophyll a, Z4 optimized chlorophyll b and total chlorophyll, and Z10 achieved the highest carotenoid levels. Yield indicators for Z4 showed a 47.94% increase in individual fruit weight and a 247.02% increase in yield per plant. Z5 increased soluble sugars, vitamin C and flavonoids by 111.4%, 68.9% and 82.0% over CK. Z6 attained peak sugar-acid ratio and anthocyanin, with respective increases of 137.3% and 56.32%. Total phenolics rose by merely 5.7% in Z3. Pearson correlation heatmap and principal component analysis (PCA) revealed significant positive correlations of yield with fruit weight, fruit-set rate and sugar content, whereas the root-to-shoot ratio was significantly negatively correlated with yield traits. The first two principal components, PC1 (growth–yield–quality axis) and PC2 (pigment–metabolism axis), collectively accounted for 64.53% of the total variation. Z4 demonstrated the optimal overall performance, attaining first position in yield per plant and second position on the flavour and quality axis (PC2), and exhibiting a balanced root-to-shoot ratio. Although Z7 achieved the highest score on PC1, this was primarily attributable to excessive vegetative growth and a relatively high root-to-shoot ratio, rather than an increase in reproductive yield.

## 1. Introduction

*Agaricus bisporus*, a typical lignocellulosic fungus, is one of the most widely cultivated edible mushrooms globally [1,2,3]. During its cultivation process, it produces considerable quantities of spent mushroom substrate (SMS) as a byproduct, a fact that has been thoroughly documented in studies focusing on mushroom cultivation and agricultural waste management [4,5,6,7]. This SMS composed of decomposed agricultural residues like straw and manure, is rich in organic matter, humic acids, essential nutrients (N, P, K), and diverse microbial communities [8,9]. It holds significant potential as an organic fertilizer and soil conditioner, capable of improving soil structure, fertility, and microbial activity [10,11,12]. With the rapid expansion of the mushroom industry, environmentally sound management of SMS waste is crucial to avoid pollution from uncontrolled disposal [13,14]. Concurrently, intensive agriculture faces challenges such as soil degradation caused by over-reliance on chemical fertilizers. Utilizing SMS as a soil amendment aligns with the need for sustainable agricultural practices [15,16].

Tomatoes are a globally important vegetable crop, valued for their nutritional content, including vitamins, minerals, and bioactive compounds like lycopene [17,18]. Most research on the reuse of spent mushroom substrate (SMS) in tomato cultivation has focused on its application as a seedling substrate or component of soilless culture mixes. [19,20]. Such research has been further advanced via systematic experiments and multi-dimensional performance assessments, with core findings centering on how SMS from different mushroom species, varied mixing ratios, and application methods affect tomato seedling development, field yield, fruit quality, and soil properties [21,22]. These studies not only verify that SMS can serve as a viable alternative to commercial seedling substrates and organic fertilizers but also uncover its unique advantages in boosting tomato plant vigor, inducing disease resistance, and optimizing nutrient supply, while clarifying the key technical points for its practical application [23,24,25,26]. Applying *Agaricus bisporus* SMS directly to soil for tomato production could simultaneously address waste management and enhance crop performance [27,28]. However, systematic studies on the effects of *Agaricus bisporus* SMS on tomato growth, yield, and quality remain limited. This study aims to fill this gap by evaluating different SMS application rates.

## 2. Materials and Methods

### 2.1. Experimental Materials

*Agaricus bisporus* spent mushroom substrate was collected from the experimental base of Jilin Agricultural University. Fully decomposed by secondary aerobic fermentation during *Agaricus bisporus* cultivation, the SMS consisted of corn straw, cow dung, peanut cake, gypsum and casing soil. The SMS was left to dry in the air, then crushed and sieved to ensure a uniform particle size for mixing with the soil and for the roots to absorb nutrients. The experimental garden soil was loam, it was sampled from a 20 cm depth in farmland, naturally air-dried, and sieved with the same 2–3 mm sieve to remove large particles such as stones and roots for homogenization. The basic physicochemical properties of the garden soil used in the experiment were as follows, bulk density 1.15 g·cm^−3^, pH 7.36, EC 0.36 mS·cm^−1^, organic matter 25.10 g·kg^−1^, ammonium nitrogen 79.05 mg·kg^−1^, available phosphorus 78.40 mg·kg^−1^, and available potassium 67.78 mg·kg^−1^.The physicochemical properties of *Agaricus bisporus* spent mushroom substrate (SMS) were as follows: bulk density 0.25 g·cm^−3^, pH 7.49, electrical conductivity (EC) 2.09 mS·cm^−1^, organic matter 384.59 g·kg^−1^, ammonium nitrogen 315.64 mg·kg^−1^, available phosphorus 512.50 mg·kg^−1^, and available potassium 1919.50 mg·kg^−1^.

Plastic buckets with a diameter of 39 cm and a height of 38 cm, and a nominal volume of approximately 45 L, were utilised as potting containers. A total of nine drainage holes, each with a diameter of 0.5 cm, were meticulously drilled in a symmetrical cross pattern at the base of each bucket. The positioning of the holes was such that one was situated at the centre, with an additional two holes at the top, bottom, left, and right. This method was employed to ensure optimal substrate permeability and to mitigate the potential for waterlogging-induced damage.

The test crop was the tomato cultivar (*S. lycopersicum* L. ‘Fenguan 888’), which exhibits resistance to root-knot nematodes, strong heat tolerance, and disease stability under high temperature and humidity conditions. It has a significantly lower incidence of early and late blight compared to conventional cultivars. As an early-maturing variety, it demonstrates excellent low-temperature adaptability, capable of normal fruiting at a daily average temperature of 12–15 °C. The fruits have a rapid enlargement rate, dense texture, sweet and sour flavor, and good transport resistance, making them suitable for long-distance fresh marketing.

### 2.2. Experimental Design and Management

A randomised experiment was conducted in a pot, using conventional garden soil as the growth substrate. The experiment comprised a total of 11 experimental treatments. A pot experiment was conducted with 11 treatments. A control and 10 treatments with SMS replacing soil at volume increments of 10% (from 10% to 100% SMS).

Each treatment comprised 10 independent biological replicates (*n* = 10), with one tomato plant grown in each pot, making a total of 110 pots. Each pot constituted an independent experimental unit. For destructive sampling, one plant was harvested from each of five randomly selected replicate pots within each treatment, whilst the remaining pots in each treatment were used for non-destructive growth measurements and fruit yield determinations. All measurements were performed in triplicate for each sample, and the data are presented as the mean of the biological replicates.

The seeds were sown on 25 March 2025 at a depth of approximately 0.8 cm in a 72-cell tray (4 × 4 × 5.5 cm), which was filled with a commercial peat–vermiculite–perlite (3:1:1) medium. The seedlings were cultivated in a solar greenhouse, with irrigation performed at intervals of 1–2 days, and a process of thinning was employed to ensure the maintenance of a single seedling per cell. Subsequent to a period of approximately 54 days, uniform seedlings (comprising 5–6 true leaves, with a length of 15–20 cm) were transplanted into experimental pots on 18 May 2025 (Table 1).

### 2.3. Determination of Tomato Growth Parameters

Tomato plant height and stem diameter were measured at 21-day intervals following transplantation, corresponding to the seedling, flowering, initial fruiting, and full fruiting stages. Plant height was measured with a tape measure from the stem base to the topmost leaf growing point, to an accuracy of 0.1 cm. Stem diameter was measured 1 cm above the cotyledon node using a vernier caliper; three measurements were taken at different stem angles, and the average was recorded (accuracy: 0.01 cm).

At the full fruiting stage, biomass (fresh and dry weights of aboveground and underground parts), root length, and root-shoot ratio (dry weight basis) were determined. For biomass measurement, tomato plants were carefully uprooted; root-attached soil was gently rinsed off with deionized water and blotted dry with filter paper to remove surface moisture. The plants were then cut into aboveground parts (stems, leaves, flowers, fruits) and underground parts (roots), and the fresh weight of each part was immediately measured with an electronic balance (accuracy: 0.01 g).

**Figure 1:**
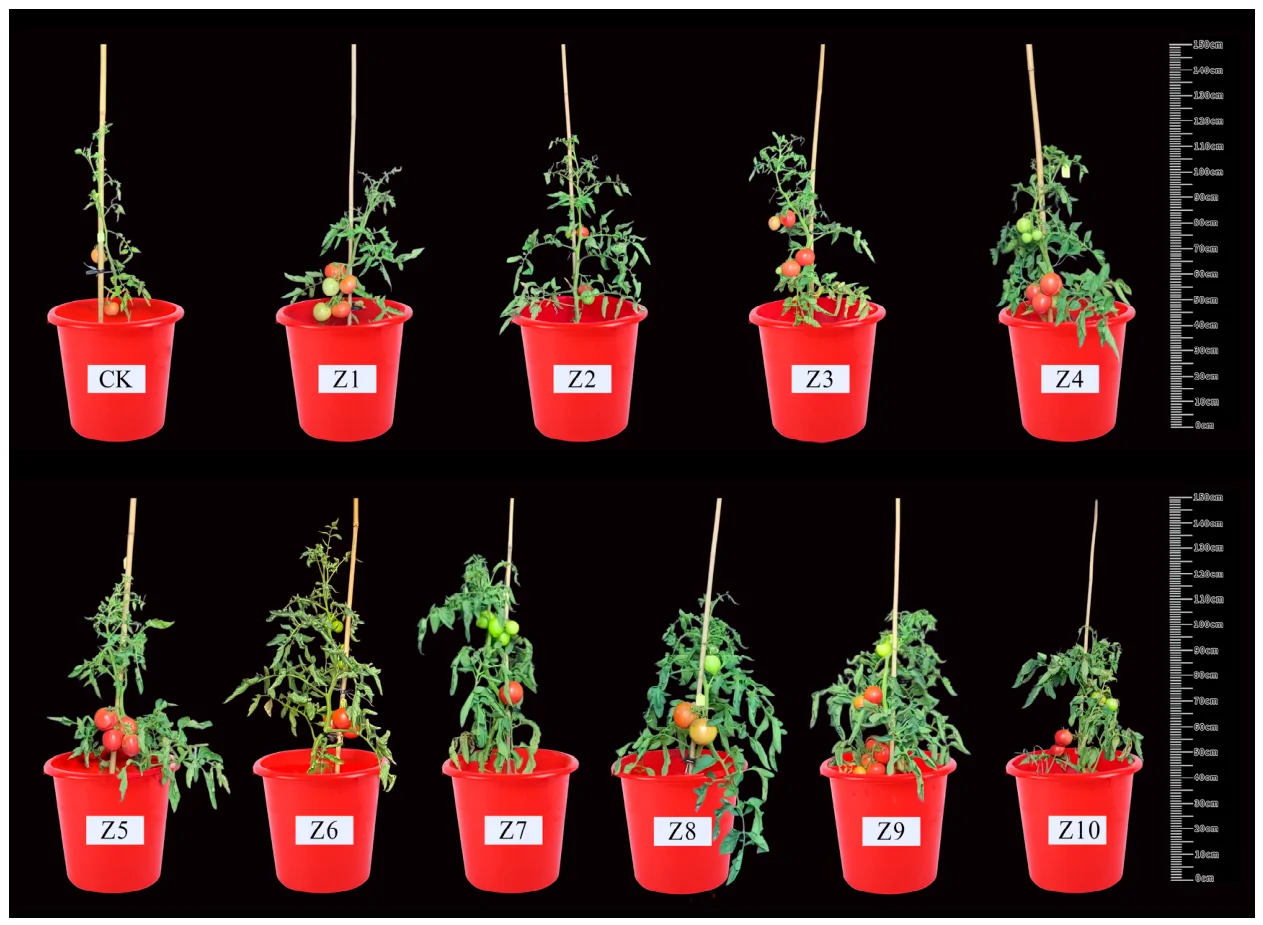
Effects of different mixing ratios of *Agaricus bisporus* spent mushroom substrate and garden soil on tomato plants.

After fresh weight measurement, aboveground and underground parts were blanched in an oven at 105 °C for 30 min, then dried at 80 °C to constant weight; the dry weight of each part was measured with the same balance. Root length was measured with a ruler from the root tip to the root collar (root-stem junction) with an accuracy of 0.1 cm. Root-shoot ratio was calculated as the ratio of the underground part dry weight to the aboveground part dry weight (Figure 1).

### 2.4. Determination of Photosynthetic Pigments at Fruiting Stage

At the initial fruiting stage, the third functional leaf of 3 tomato plants per treatment in each plot was selected for sampling. A 0.2 g sample of tomato leaves was placed into a test tube containing 20 mL of an ethanol-acetone mixed solution (volume ratio 1:1) and kept in a dark environment for 24 h [29]. After that, a spectrophotometer (UV-2450, Shimadzu, Tokyo, Japan) was used to determine the absorbance of the extracted pigments at wavelengths of 470 nm, 649 nm, and 665 nm [30]. The concentrations of chlorophyll a, chlorophyll b, and carotenoids were then calculated according to the previously described method. Each treatment was repeated three times.

### 2.5. Determination of Fruit Morphological Indicators

**Figure 2:**
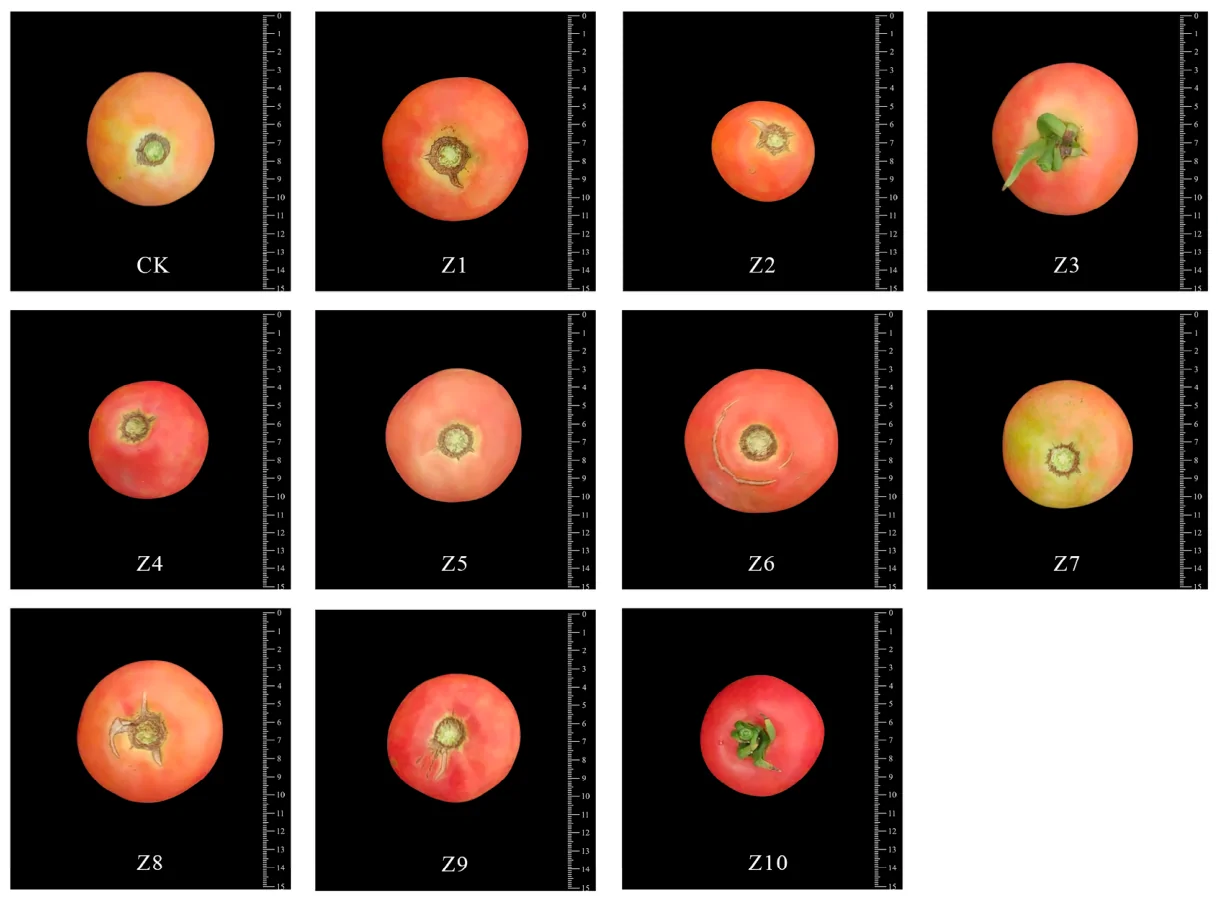
Tomato fruits under different ratios of *Agaricus bisporus* spent mushroom substrate and garden soil.

The selected fruits mentioned above were used for the determination of the samples. A vernier caliper (CD-15AX, Mitutoyo Corporation, Tokyo, Japan) and an electronic balance (JY1002, Shanghai Jiesheng Scientific Instruments Co., Ltd., Shanghai, China) were used to measure fruit morphological indicators with accuracies of 0.01 mm and 0.01 g, respectively, and each treatment was repeated 3 times. The fruit shape index was calculated as longitudinal diameter/transverse diameter. A GY-4 series digital display fruit hardness tester (Boshi Electronic Instruments, Hangzhou, China) was used to measure fruit hardness, with 3 repetitions per treatment [31] (Figure 2).

### 2.6. Determination of Tomato Yield

During the maturity stage, the yield of tomato plants in each treatment was measured and recorded with an electronic balance with an accuracy of 0.01 g per harvest. The total yield of tomato plants in each treatment was calculated cumulatively, with three repetitions per treatment.

### 2.7. Determination of Fruit Quality-Related Biochemical Indicators

Pulp samples prepared using the experimental methods described above were used to determine the fruit quality indicators.

The soluble solids content was directly measured using a digital refractometer (RHBO-90 Handheld, Link-Co. Ltd.,New Taipei City, Taiwan), following the method described by Kayikci et al. [32].

For the determination of soluble sugar content, 0.1 g of the sample was accurately weighed and transferred into 50 mL of water, followed by boiling for 1 h to prepare the reaction solution, and the determination method referred to the protocol described by Li et al. [33].

The titratable acid content was determined by weighing 3 g of the sample and mixing it thoroughly with 50 mL of water to prepare the reaction solution. The organic acid content was then measured via acid–base titration. The sugar–acid ratio was calculated by dividing the soluble sugar content by the titratable acid content, using the method described by Zhao, Wu, Wu, Yu, Cao and Ma [17].

In order to determine the vitamin C (Vc) content, 3 g of the sample was weighed and extracted with 5 mL of oxalic acid solution. The Vc content was then determined using the molybdenum blue colorimetric method, as described by Mellidou et al. [34].

The sugar–acid ratio was calculated as the ratio of soluble sugar content to titratable acid content. Both soluble sugar content and titratable acid content were determined according to the methods described above, respectively [35]. All measurements were performed in three biological replicates per treatment, in order to ensure the accuracy and reliability of the experimental data.

The total phenolics and flavonoid contents were determined using the method described by Singleton et al. [36]. A standard curve for total phenols was prepared using gallic acid as the standard. The sample should be weighed at 0.5 g, and 3 mL of 95% ethanol was added. The sample was then extracted by shaking at 60 °C for 2 h. After extraction, the sample was centrifuged at 12,000 rpm at 25 °C for 10 min, the resulting pellet was collected, and it was diluted to 3 mL with 95% ethanol. Following reaction with Folin’s reagent (phenol reagent, Beijing Solarbio Science & Technology Co., Ltd., Beijing, China) was used. absorbance was measured at a wavelength of 760 nm. For the flavonoid standard curve, rutin was used as the standard. A 1 g sample was weighed and extracted in the dark with 20 mL of a methanol solution containing 1% hydrochloric acid to prepare the test solution. After reaction with aluminum nitrate and sodium hydroxide, the absorbance of the test solution was measured at 510 nm.

Soluble protein content was determined using the Coomassie brilliant blue staining method. A 0.5 g sample of fruit pulp was weighed and homogenized with 5 mL of phosphate buffer (pH 7.8). The mixture was centrifuged at 8000 rpm and 4 °C for 15 min, and the supernatant was taken as the crude extract. Then, 0.1 mL of the crude extract was mixed with 5 mL of Coomassie Brilliant Blue G-250 reagent (Beijing Solarbio Science & Technology Co., Ltd., Beijing, China), allowed to stand at room temperature for 5 min, and the absorbance was measured at 595 nm. The soluble protein content was calculated according to the standard curve established with bovine serum albumin (BSA) [37,38].

The following investigation focuses on the content of anthocyanins. The relative content of anthocyanins was determined by spectrophotometry. Briefly, 200 mg of fruit pulp sample was weighed and extracted with 5 mL of acidic methanol containing 1% HCl, followed by shaking and incubating at room temperature for 18 h. After extraction, the mixture was centrifuged, and the absorbance of the supernatant was measured at wavelengths of 530 nm and 657 nm using a spectrophotometer. The relative content of anthocyanin was calculated by the formula (A530 − 0.25 × A657) g^−1^ FW. Each group of data was repeated three times to ensure the reliability of the experimental results. Wherein, A530 and A657 represent the absorbance values of the extract at 530 nm and 657 nm, respectively; FW refers to the fresh weight of the sample [39]. Each group of data was repeated three times to ensure the accuracy and repeatability of the experimental data.

### 2.8. Statistical Analysis

All parameters were analyzed at least in triplicate, and results were expressed as mean ± standard deviation (SD). Data were processed using Excel 2019. One-way analysis of variance (ANOVA) was conducted, followed by the Duncan multiple range test, utilising SPSS 26.0 software. A significance level of (*p* < 0.05) was employed to determine statistical significance. Pearson’s correlation coefficient and principal component analysis. The component analysis of all graphs was conducted utilising Origin 2021.

## 3. Results and Analysis

### 3.1. Effects on Tomato Growth

**Figure 3:**
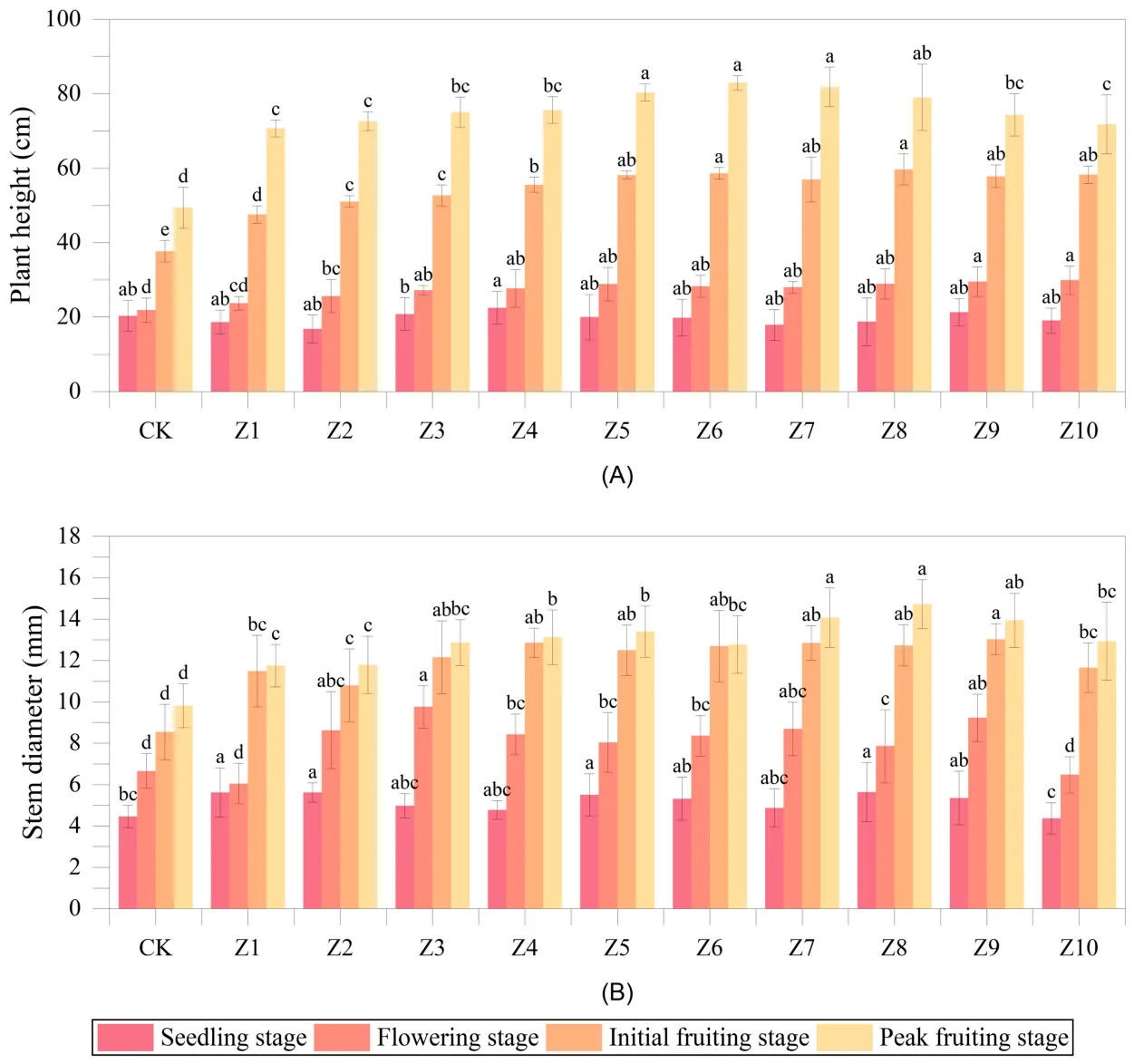
Effects of *Agaricus bisporus* Spent Mushroom Substrate on tomato growth indicators under different treatments at the full fruiting stage. (**A**) Plant height, (**B**) Stem diameter. Different lowercase letters are expressed as the same period at the 0.05 level.

As shown in Figure 3A,B, in all treatment groups, tomato plant height and stem diameter followed a trend of first increasing and then decreasing from the seedling stage to the peak fruiting period. Within each growth stage, there were significant differences in these parameters among the different treatment groups, as indicated by the different lowercase letters labeled on the bar charts (*p* < 0.05). Throughout the entire growth period, the promoting effect of SMS on tomato morphological traits gradually increased.

At the seedling stage, mushroom substrate treatments significantly increased stem diameter, with the Z1 showing the most obvious effect, increasing stem diameter by 26.2% relative to the control, while no significant differences in plant height were detected between the treatment groups and the control group.

At the flowering stage, mushroom substrate application significantly improved plant morphological indices and exerted positive effects on plant height and stem diameter in all subsequent growth stages. Z10 achieved the maximum plant height, with an increase of 36.4% compared with the control, whereas Z3 resulted in the thickest stem diameter, representing an increase of 46.5% relative to the control.

At the initial fruiting stage, the tallest plants were found in Z8, with a 58.5% increase over the control, and the thickest stems were recorded in Z9, with an increase of 52.6% compared with the control. At the peak fruiting stage, the Z6 produced the maximum plant height throughout the growth cycle, which was 68.0% higher than that of the control, and the Z8 showed the thickest stem diameter, with an increase of 50.0% relative to the control. In summary, these results indicate that mushroom substrate treatments significantly promote tomato plant growth during the middle and late growth stages, and the average values of plant height and stem diameter at the peak fruiting stage are increased by 54.4% and 34.8%, respectively, compared to the control.

Table 2 provides an evaluation of the impacts of various treatments on plant growth, as measured by biomass accumulation and distribution. The application of SMS resulted in a substantial enhancement of biomass accumulation, both in aboveground and belowground plant components. Z9 resulted in a significant increase in aboveground fresh weight, with a relative increase of 580.7% compared to CK, representing the most substantial gain in this index. Z8 resulted in the greatest increases in aboveground dry weight and belowground fresh weight, with respective increases of 163.9% and 312.8% compared to the control group. All treatment groups exhibited significantly lower root-to-shoot ratios in comparison to CK. Furthermore, Z9 induced the most significant increase in root length, with a 63.9% increase in comparison with the control.

### 3.2. Effects on Photosynthetic Pigments in Leaves

**Figure 4:**
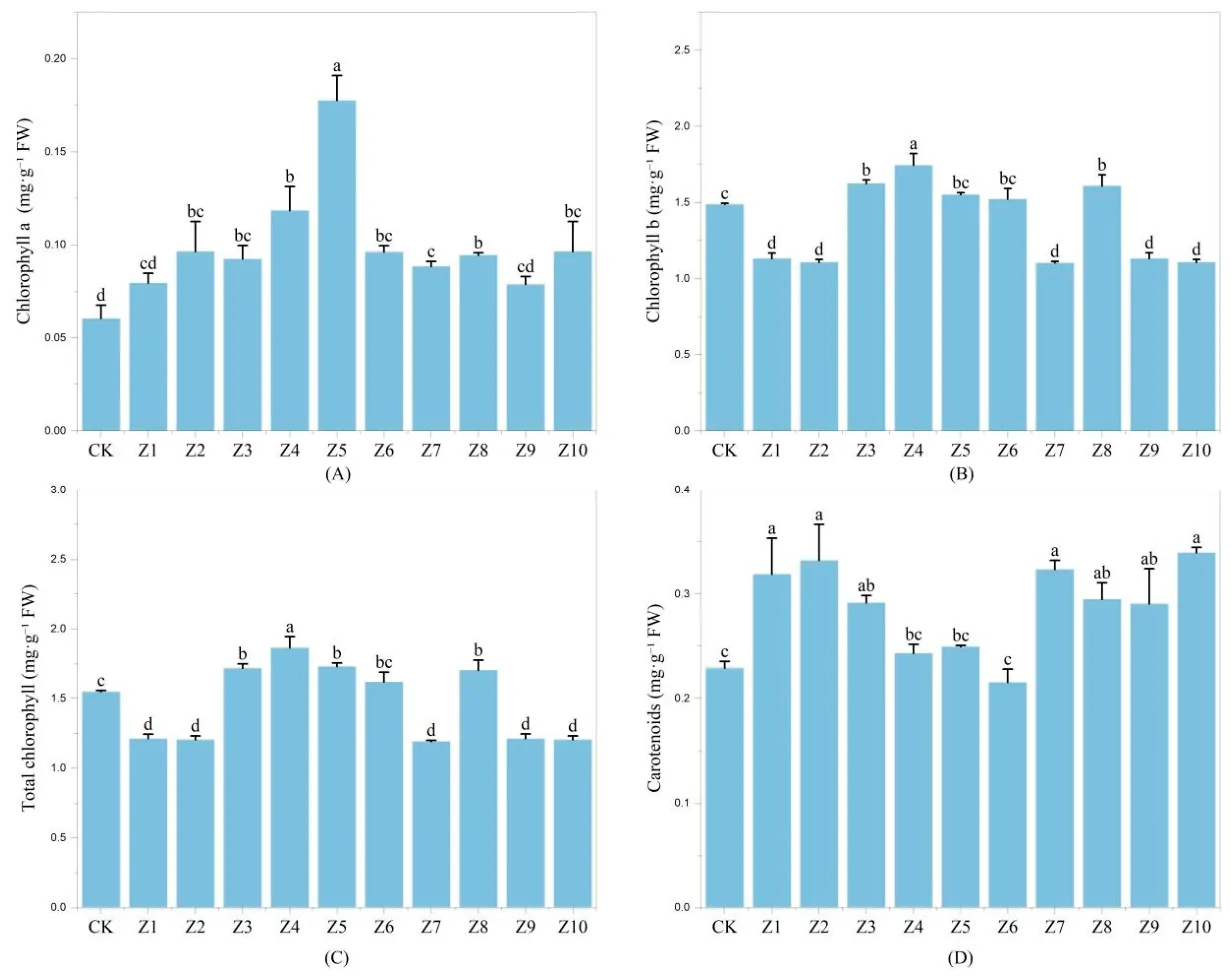
Effects of *Agaricus bisporus* SMS on leaf photosynthetic pigments of tomato plants. (**A**) Chlorophyll a, (**B**) Chlorophyll b, (**C**) Total chlorophyll concentrations, and (**D**) Carotenoids. illustrates the differences in pigment contents across all treatment groups, with error bars representing standard error (SE) and letters indicating statistical significance (*p* < 0.05).

Figure 4A–D illustrates the changes in chlorophyll a, chlorophyll b, total chlorophyll, and carotenoid contents in the leaves of plants from the different treatment groups (Z1–Z10) and the CK.

As illustrated in Figure 4A, chlorophyll a content exhibited an initial increase followed by a decrease as treatment intensity increased, reaching a peak under Z5 (0.178 mg/g FW), representing a significant increase of 194.07% compared to CK (0.060 mg/g FW).

As illustrated in Figure 4B, chlorophyll b and total chlorophyll content similarly exhibited a pattern of initially increasing and then decreasing with increasing treatment intensity; both reached their highest levels under Z4, at 1.745 mg/g FW and 1.864 mg/g FW, respectively, representing increases of 17.39% and 20.46% compared to CK; whereas chlorophyll b content was lowest under Z7 (1.101 mg/g FW), representing a decrease of over 25% compared to CK.

As illustrated in Figure 4C, total chlorophyll content was lowest under Z7 (1.190 mg/g FW), a decrease of 22.99% compared to CK, and all these differences were statistically significant (*p* < 0.05).

As illustrated in Figure 4D, carotenoid content was highest under Z10 (0.339 mg/g FW), representing a 48.14% increase compared to CK, and lowest under Z6 (0.215 mg/g FW), representing a 5.99% decrease compared to CK; both differences were statistically significant compared to the control (*p* < 0.05).

### 3.3. Effects on Fruit Morphology

As illustrated in Table 3, SMS significantly altered the shape parameters of tomato fruits. Among these, Z8 was the most effective in increasing fruit size, with the fruit’s diameter and length increasing by 25.7% and 20.1%, respectively, compared to CK. However, this treatment also led to a decrease in fruit firmness, with Z7 exhibiting the lowest firmness, a significant reduction of 24.6% compared to CK. There were no significant differences in the fruit shape index among the treatment groups compared to CK. In summary, SMS effectively promotes fruit enlargement, but some treatments are accompanied by a decrease in fruit firmness.

### 3.4. Effect on the Amount of Fruit Produced

As illustrated in Table 4, the appropriate SMS application significantly enhanced tomato yield-related indicators, exhibiting a rise-and-fall trend. Z4 performed best in terms of individual fruit weight and yield per plant, with average individual fruit weight and yield per plant significantly increasing by 47.94% and 247.02%, respectively, compared with the control group. At the same time, all treatments with SMS significantly increased the number of set fruits, with Z5 and Z8 yielding the highest numbers, representing a significant increase of 155% compared to CK. Furthermore, the Z10 produced the highest number of fruit clusters, showing a significant increase of 78.9% compared to the CK. In summary, the application of SMS achieved a substantial increase in yield per plant through the synergistic increase in individual fruit weight, fruit set, and the number of fruit clusters, with Z4, Z3, and Z8 demonstrating the most notable performance in individual fruit weight and yield per plant.

### 3.5. Effects on Fruit Quality Indicators

A significant divergence in fruit quality parameters was observed between the treatment group and the control. These parameters exhibited an initial increase, followed by a subsequent decrease, in accordance with the SMS application rate.

**Figure 5:**
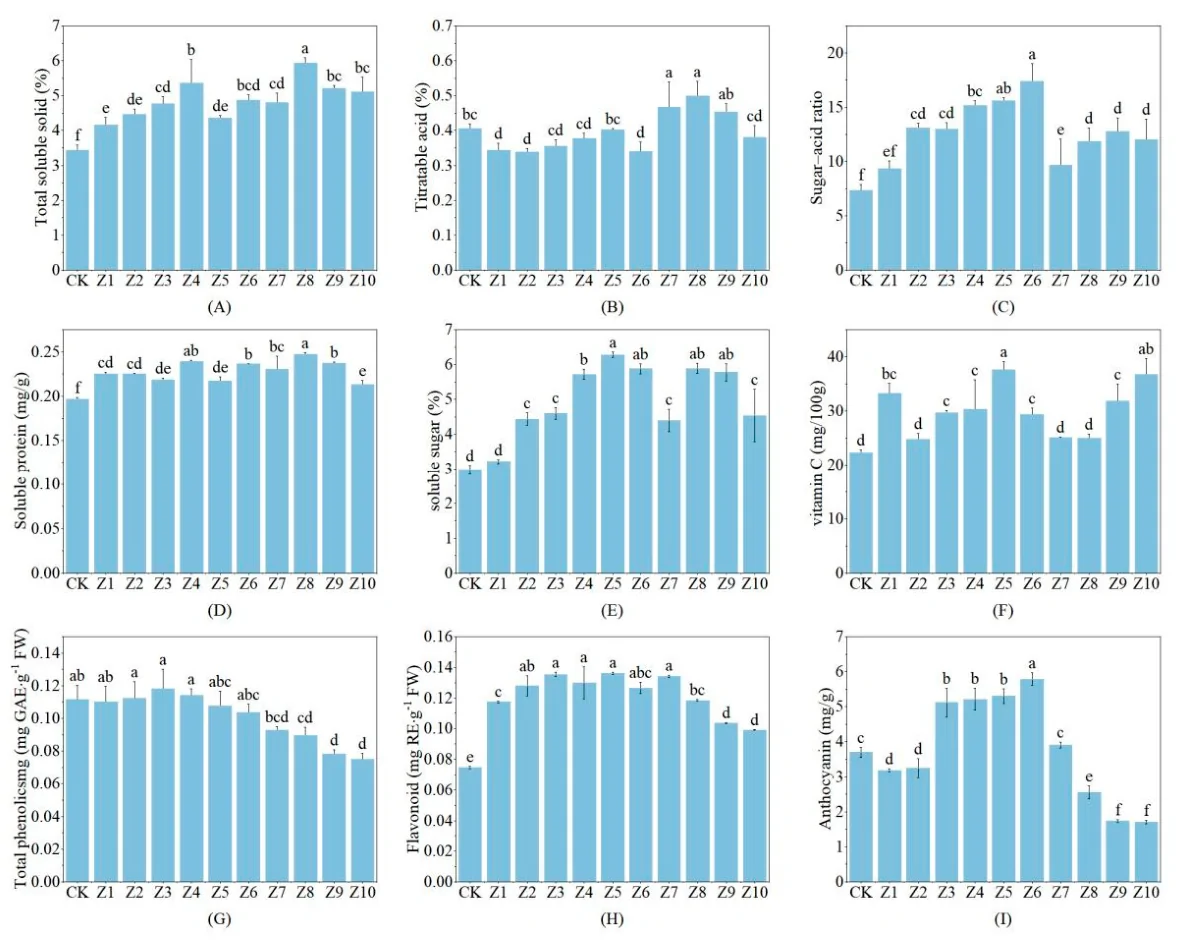
Effects of *Agaricus bisporus* SMS on contents of primary metabolites and bioactive compounds in tomato fruit. (**A**) Total soluble solids, (**B**) Titratable acid, (**C**) Sugar–acid ratio, (**D**) Soluble protein, (**E**) Soluble sugar, (**F**) Vitamin C, (**G**) Total phenolics, (**H**) Flavonoids, and (**I**) Anthocyanins. Different lowercase letters are expressed as the same period at the 0.05 level.

As demonstrated in Figure 5A, soluble solids content (SSC) presented a rising-then-falling trend along the SMS gradient and was maximized under Z8, which was a significant 72.8% increase compared with the CK and significantly higher than that of other treatments.

As demonstrated in Figure 5B, the content of titratable acid (TA) increased initially and then decreased in proportion to the increasing SMS gradient. Z8 achieved the highest value, showing a significant 23.1% increase compared with the CK; in contrast, Z6 only had a TA content that was a 16.3% decrease relative to CK.

As demonstrated in Figure 5C, sugar–acid ratio (SAR) varied dynamically with SMS gradient following an increasing–decreasing pattern. Z6 exhibited the highest SAR, representing an increase of 137.3% relative to CK. In contrast, CK had the lowest SAR.

As demonstrated in Figure 5D, soluble protein content showed an initial increase and subsequent decrease in response to elevated SMS gradient, with the maximum at Z8, indicative of a substantial 25.9% increase in relation to CK.

As demonstrated in Figure 5E, soluble sugar content exhibited an initial increase followed by a subsequent decrease with increasing SMS, reaching its maximum at Z5 with a significant 111.4% increment compared with CK.

As demonstrated in Figure 5F, vitamin C content conformed to the universal rising-then-falling trend across the SMS gradient. Z5 demonstrated optimal performance, exhibiting a substantial 68.9% increase relative to CK.

As demonstrated in Figure 5G, the total phenolics content exhibited an initial increase, followed by a subsequent decrease with increasing SMS application rates, reaching a peak at Z3. The total phenolics content in Z3 reached its maximum, which was 5.7% higher than that in CK. The total phenolics content exhibited a notable elevation in Z2, Z3 and Z4, with values that were considerably higher than those observed in Z7–Z10.

As demonstrated in Figure 5H, flavonoid content increased rapidly with rising SMS amendment rates and then declined at higher application rates. Under Z5, flavonoid content reached its maximum, representing a significant 82.0% increase relative to CK.

As demonstrated in Figure 5I, the content of anthocyanin exhibited a rapid increase with rising SMS amendment rates, subsequently declining at higher application rates. Z6 achieved the maximum anthocyanin content, representing an increase of 56.32% relative to CK.

Collectively, most treatments improved tomato fruit quality relative to CK, with the Z5 and Z8 treatments demonstrating the best comprehensive performance. Z5 showed significant advantages in Vc, flavonoid, and soluble sugar contents, while the Z8 excelled in SSC and soluble protein contents. In contrast, Z9 and Z10 performed relatively poorly in total phenolics, flavonoid, and anthocyanin contents, with values that were significantly lower than those of the CK.

### 3.6. Principal Component Analysis

**Figure 6:**
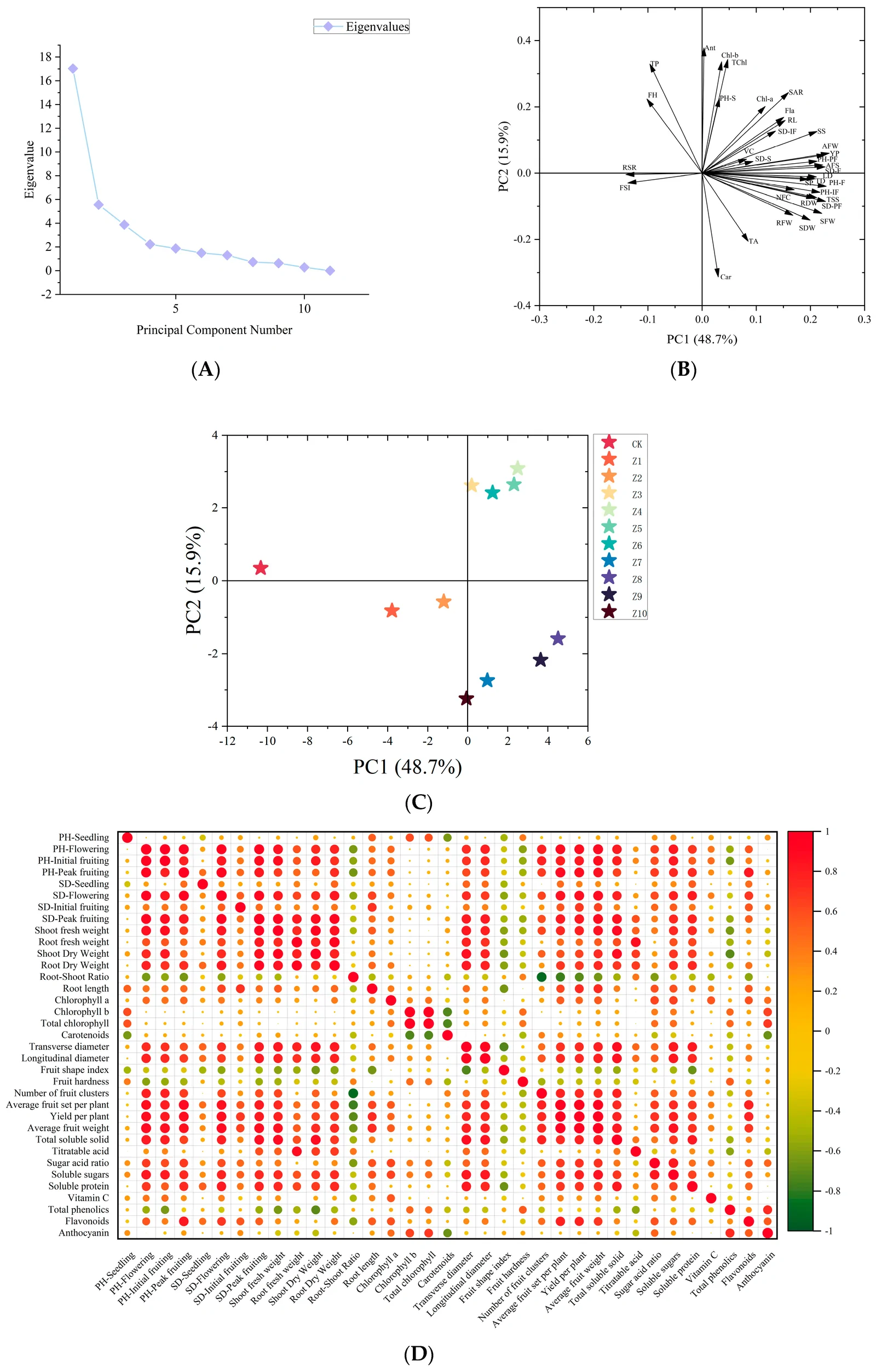
PCA and Correlation Heatmap of the Effects of *Agaricus bisporus* Spent Mushroom Substrate Application on Tomato Yield and Quality. (**A**) Eigenvalues; (**B**) Loadings; (**C**) PC scores; (**D**) Heatmap. Correlations were assessed using Pearson’s correlation analysis (*p* < 0.05). Abbreviations of indicators in panel (B) refer to Table S1.

Principal component analysis (PCA) and Pearson correlation analysis were performed on 35 traits relating to tomato growth, yield and quality; the results are shown in Figure 6. As illustrated in Figure 6A, the eigenvalue plot is presented, while Figure 6B details the factor loading plot. Figure 6C shows the sample score plot, and Figure 6D presents the correlation heatmap (*p* < 0.05).

As shown in Figure 6A, a total of seven principal components were extracted according to the Kaiser criteria (eigenvalues > 1), cumulatively explaining 95.31% of the total variance. The scree plot shows a distinct inflection point after PC3, with the eigenvalues declining rapidly from PC1 to PC3 before levelling off. PC1, PC2 and PC3 explain 48.65%, 15.88% and 11.07% of the total variance respectively, with the three combined accounting for 75.60%.

As shown in Figure 6B, PC1 is primarily composed of positive loadings for yield per plant (YP), average fruit weight (AFW), plant height at flowering (PH-F), stem diameter at peak fruiting (SD-PF), stem diameter at flowering (SD-F), number of fruits per plant (AFS), fresh weight of aboveground parts (SFW), plant height at the beginning of fruiting (PH-IF) and soluble sugars (SS); whilst the root-to-shoot ratio (RSR), fruit shape index (FSI), fruit firmness (FH) and total phenolics (TP) represent negative loadings, reflecting a gradient from ‘low yield with high root-to-shoot ratio’ to ‘high yield with vigorous growth’. In PC2, positive loadings were observed for carotenoids (Car), titratable acidity (TA), fresh dry weight of aboveground parts (SDW) and fresh root weight (RFW), whilst negative loadings were observed for anthocyanins (Ant), total chlorophyll (TChl), chlorophyll b (Chl-b) and the sugar-to-acid ratio (SAR), representing variation in photosynthetic pigment composition and secondary metabolite accumulation. PC3 is primarily associated with variation in chlorophyll a (Chl-a), vitamin C (Vc) and soluble protein (SP), representing an axis of antioxidant and nutritional quality that is independent of yield.

As shown in Figure 6C, the various fungal residue treatments can be clearly distinguished on the PCA plot. Treatments with higher application rates are positioned further to the right of PC1, corresponding to higher yields, larger individual fruits and more vigorous plant growth; whereas the control and low-application-rate treatments are concentrated to the left of PC1, with a higher root-to-shoot ratio and lower overall yields. Differences along the PC2 axis primarily reflect variations in the composition of photosynthetic pigments and the content of secondary metabolites across the different treatments.

As shown in Figure 6D, there are complex correlations amongst the 35 traits. Amongst these, traits related to yield exhibit strong positive correlations; for example, there is a high correlation between yield per plant and fruit weight (r = 0.979) as well as between yield per plant and the number of fruits set per plant (r = 0.978), indicating that yield is primarily determined by the combined effects of fruit number and fruit weight. Furthermore, strong correlations were observed between growth traits at different growth stages; for example, plant height at flowering correlated strongly with plant height at the onset of fruiting (r = 0.958), and stem diameter at flowering correlated strongly with stem diameter at peak fruiting (r = 0.913), suggesting that growth trends remain consistent across all growth stages. Furthermore, the root-to-shoot ratio showed the most significant negative correlation with reproductive traits; specifically, it exhibited a clear negative correlation with the number of fruit clusters (r = −0.872), the number of fruits per plant (r = −0.760) and yield per plant (r = −0.718), indicating that an excessively high root-to-shoot ratio has an adverse effect on reproductive growth. Furthermore, soluble sugars (r = 0.788), soluble solids (r = 0.772) and flavonoids (r = 0.775) all exhibited strong positive correlations with yield per plant, suggesting that the fungal residue not only increased yield but also improved nutritional quality; in contrast, the correlation between vitamin C and most yield traits was relatively weak, indicating that its accumulation is regulated by independent pathways.

## 4. Discussion

The present study systematically evaluated the effects of *Agaricus bisporus* spent mushroom substrate on the growth, yield, and fruit quality of tomato cultivar (*S. lycopersicum* L. ‘Fenguan 888’). The findings indicated that the judicious deployment of SMS application could substantially enhance tomato vegetative growth, improve yield components, and optimise fruit nutritional quality. Conversely, an excess of SMS has been demonstrated to exert a reciprocal effect, resulting in diminished tomato yield and compromised fruit quality. The underlying principle pertains to the direct impact of SMS application rates on tomato growth, metabolic processes, nutrient allocation, and the equilibrium between nutrients and secondary metabolites.

Whilst the proportion of SMS was increased, changes also occurred in nutrient supply, organic matter content, salinity (electrical conductivity) and the physical properties of the growing medium. Consequently, the observed growth response cannot be attributed solely to SMS as a nutrient source. The specific contributions of each factor require further investigation.

### 4.1. Regulatory Mechanisms of Agaricus bisporus Spent Mushroom Substrate on Tomato Growth

Plant height and stem diameter are key indicators for evaluating tomato vegetative growth and overall plant vigor [40,41]. Previous studies have demonstrated that the application of SMS has a consistent effect on the morphological development of tomatoes [21,42]. In this experiment, tomato seedlings exhibited a relatively weak response to SMS during the early seedling stage, with only slight improvements observed in some morphological parameters. However, as the plants entered the middle and late growth stages, the growth-promoting effects of SMS became more pronounced, consistent with the findings of previous studies. The nutrients contained in SMS are not immediately available for plants, as they must undergo mineralization and conversion before being effectively absorbed and utilised [19,43,44]. This process is closely linked to the growth progression and physiological state of the tomatoes [45]. As the growing season progressed, plants exhibited stronger metabolism and nutrient utilisation, and the growth-promoting effect of SMS became increasingly evident, significantly enhancing tomato vigour [46]. SMS has been demonstrated to enhance plant productivity, exhibiting a concentration-dependent improvement over time. Its slow nutrient release has been shown to promote sustained growth and biomass accumulation [47]. The findings of this study were further corroborated by correlation analysis, which provided additional evidence to support the temporal effect observed. Growth traits exhibited a strong positive correlation at varying growth stages, indicating that early plant vigour can predict subsequent growth and yield potential. This provides a theoretical basis for evaluating the efficacy of SMS in tomato cultivation.

The SMS application has been demonstrated to regulate root physiology and may enhance and promote biomass accumulation [48]. This experiment shows that moderate levels of SMS exert a substantial positive influence on the accumulation of above-ground and below-ground biomass in tomato plants. Conversely, excessive amounts of SMS have been demonstrated to result in a reduction in biomass accumulation. Following the improvement of nutrient availability, the roots no longer require a significant investment of carbon sources for foraging, thus allowing for greater allocation of photosynthates to aboveground growth [45,49]. This, in turn, facilitates the coordinated development of vegetative and root growth, thereby enhancing the plant’s capacity for resource capture and utilisation. However, excessive SMS addition reduced tomato biomass accumulation [7]. High application rates altered medium physical properties, disturbed root development, and increased electrical conductivity, inducing salt stress and reducing nutrient uptake [50]. Intensive microbial decomposition of excess organic matter may also immobilize nitrogen, suppressing plant growth [51]. A robust negative correlation has been observed between the root-to-shoot ratio and yield traits. It has been demonstrated that, at elevated levels of SMS application, an excessively elevated root-to-shoot ratio gives rise to an excessive allocation of nutrients to the root system, thereby impeding reproductive growth. This finding suggests that there exists an optimal balance between root and shoot growth, which is necessary to achieve maximum productivity, which is in agreement with Wang et al. [52].

Photosynthetic pigments are vital for the conversion of light energy into chemical energy and thereby directly determine the photosynthetic capacity of tomato leaves [53,54]. This study examined the effects of SMS application on the chlorophyll content of tomato leaves. Moderate application increased the levels of chlorophyll a, chlorophyll b, and total chlorophyll, while excessive application had an inhibitory effect. In contrast, carotenoid content exhibited an opposite trend. This study corroborates the findings of Asemoloye et al. [55] that SMS significantly increases the content of chlorophyll a, chlorophyll b, and total chlorophyll. Conversely, an elevated SMS application ratio has been observed to elicit the plant’s innate adaptive defence response, inducing carotenoid synthesis [56]. Through the photoprotective action of carotenoids, damage to photosynthetic structures caused by intense light is mitigated, ensuring the continuous and stable functioning of photosynthesis [56].

### 4.2. Effects of Agaricus bisporus Spent Mushroom Substrate on the Pathways of Tomato Yield Formation

SMS increases tomato yields not by regulating a single trait, but by coordinating multiple yield-determining factors to achieve an overall yield improvement [57]. The present study showed that appropriate SMS improved tomato fruit size while maintaining regular fruit shape. The width of the fruit along the length and width showed an overall increase across the different treatments, and the shape index stayed at a level close to a circle. The shape and firmness of the fruit were not significantly different in most SMS treatments compared with the control, consistent with previous studies [58]. Lopes, Zied, Martos, de Souza, Da Silva and Dias [20] observed that during the cultivation of tomato seedlings, there was a significant increase in fruit yield at all stages of harvest when the proportion of SMS was increased relative to the commercial control substrate. Appropriate SMS can promote the vegetative growth of tomatoes, suggesting an increase in the production of photosynthetic compounds. This provides an ample source for yield formation. However, excessive amounts will result in reduced yields [21,59]. In addition, SMS was found to effectively promote flower bud differentiation, improve the development of reproductive organs, optimise the allocation of photosynthetic products to reproductive sinks, strengthen sink activity, and coordinate source-sink balance; this resulted in a significant increase in yield [60,61]. The regulatory effects of different SMS application rates on yield components vary; this variation may be partly attributable to differences in the extent to which each rate regulates the source-sink relationship [62]. It is hypothesised that an appropriate SMS application rate may be more conducive to achieving source-sink balance, thereby yielding more favourable yield increases [63]. The findings of the present study indicate that yield per plant is significantly positively correlated with both average fruit weight and the average number of fruits set per plant. Furthermore, a high degree of coordination is observed between these two yield components [64]. The hypothesis that SMS promotes the overall synergistic yield of reproductive development is one that merits further investigation [65]. The strong correlation between yield and plant height and stem diameter further suggests that vigorous vegetative growth during the early reproductive stage may be physiologically linked to high yields [66].

### 4.3. Key Factors of Agaricus bisporus Spent Mushroom Substrate on Tomato Quality Regulation

In tomato fruit, key indicators of quality include components such as soluble proteins, soluble sugars, soluble solids, organic acids, vitamin C, total phenolics, flavonoids, and anthocyanins. These components not only determine the flavour and nutritional value of tomatoes, but also influence their commercial value [67,68,69]. The findings of this experiment suggest that, under suitable levels of SMS, the content of soluble sugars, soluble solids, soluble proteins, and vitamin C in tomato fruits can be enhanced to various extents, with levels initially rising and then declining as the application rate of SMS increases. It is possible that an appropriate SMS has been demonstrated to achieve precise matching of tomato nutrient supply and demand, provide sufficient raw materials and energy for the synthesis of fruit nutrients and secondary metabolites, and avoid metabolic disorders caused by nutrient imbalance [70,71]. The result of these processes is the promotion of the synthesis and accumulation of beneficial nutrients, the optimisation of fruit flavour and nutritional quality, the maintenance of the balance of fruit sugar–acid ratio, and the improvement of comprehensive fruit quality [72]. Excessive application of SMS will disrupt the metabolic balance of tomatoes, leading to deterioration in fruit quality [71,73]. The fundamental issue pertains to the disruption of the harmonious interplay between primary and secondary metabolism within tomato plants, a consequence of an imbalance in nutrients resulting from excessive SMS [20]. The total phenolics compounds exhibited a significant negative correlation with the dry and fresh weights of the aerial parts. Conversely, flavonoids demonstrated a significant positive correlation with yield. However, anthocyanins exhibited only a weak correlation with both growth and yield traits. It is evident that different secondary metabolites respond in a disparate manner to SMS. Moreover, the results of the PCA demonstrate that antioxidant traits are distributed across a variety of principal components. This finding indicates that excessive SMS intake may disrupt the coordinated regulation between primary and secondary metabolism, consequently resulting in an imbalance in fruit quality.

## 5. Conclusions

This study investigated the effects of ten different application ratios of SMS on the growth, yield, and fruit quality of the tomato cultivar (*S. lycopersicum* L. ‘Fenguan 888’), and further explored the underlying regulatory mechanisms.

SMS was found to exert a stage-dependent regulatory effect on tomato growth. Evaluation of plant height revealed no discernible disparities at the seedling stage. However, a pronounced enhancement in both plant height and stem diameter was observed from the flowering to the peak fruiting stages. The application of an appropriate amount of SMS is associated with improvements in root morphology, increased photosynthetic pigment content and enhanced plant biomass accumulation. It should be noted that this study did not directly measure nutrient uptake or photosynthetic parameters; therefore, the observed growth responses are likely related to improvements in soil physicochemical properties and nutrient availability. However, the specific physiological mechanisms require further investigation.

The study found that reasonable SMS ratios were able to coordinate vegetative and reproductive growth, thereby optimising yield components. Z4 achieved the highest fruit yield per plant and the most balanced yield–quality performance. The regulatory effects of SMS on tomato fruit quality were found to be significant, with evident variations among treatments. Optimal SMS rates facilitated the accumulation of vitamin C, flavonoids, soluble sugar and soluble proteins, and maintained a desirable sugar–acid ratio. The application of excessive SMS led to an increase in titratable acid content, a disruption of the sugar–acid balance, a reduction in fruit firmness, and an impairment of flavour and antioxidant properties.

Principal component analysis indicated that the first principal component (PC1, growth–yield–biomass axis) and the second principal component (PC2, pigment–quality axis) together accounted for 64.53% of the total variation. Treatment Z4 achieved the optimal overall balance, ranking first in fruit yield, second on the PC2 quality axis, and exhibiting a balanced root-to-shoot ratio. Although the high SMS treatments (Z7, Z8) scored higher on PC1, this was primarily due to excessive vegetative growth. In summary, when *Agaricus bisporus* SMS is used as an organic soil conditioner in appropriate proportions, it not only effectively promotes tomato growth, increases yield and optimises fruit quality, but also demonstrates potential for the effective utilisation of agricultural waste. The study confirmed that Z4 is a technically feasible strategy for the sustainable cultivation of tomatoes.

## Figures and Tables

**Table 1:** The ratio of garden soil to *Agaricus bisporus* spent mushroom substrate.

Treatments	Rural Soil	*Agaricus bisporus* Spent Substrate
CK	100%	0%
Z1	90%	10%
Z2	80%	20%
Z3	70%	30%
Z4	60%	40%
Z5	50%	50%
Z6	40%	60%
Z7	30%	70%
Z8	20%	80%
Z9	10%	90%
Z10	0%	100%

**Table 2:** Effects of *Agaricus bisporus* Spent Mushroom Substrate on Biomass Accumulation and Root Architecture in Tomato Plants. Different lowercase letters are expressed as the same period at the 0.05 level.

Treatments	Shoot Fresh Weight (g)	Root Fresh Weight (g)	Shoot Dry Weight (g)	Root Dry Weight (g)	Root-Shoot Ratio	Root Length (cm)
CK	57.00 ± 27.52 ^f^	13.03 ± 1.62 ^d^	36.00 ± 5.48 ^d^	2.77 ± 0.36 ^d^	0.27 ± 0.13 ^a^	36.64 ± 9.05 ^c^
Z1	167.00 ± 48.55 ^e^	18.65 ± 5.88 ^d^	46.00 ± 8.94 ^cd^	5.53 ± 0.95 ^bcd^	0.12 ± 0.05 ^bc^	45.90 ± 4.59 ^abc^
Z2	217.00 ± 30.12 ^cde^	16.30 ± 6.88 ^d^	47.00 ± 5.7 ^cd^	5.17 ± 1.12 ^cd^	0.07 ± 0.02 ^bc^	49.66 ± 11.5 ^abc^
Z3	199.00 ± 54.36 ^de^	18.70 ± 9.55 ^d^	62.00 ± 14.83 ^bc^	5.24 ± 2.15 ^cd^	0.1 ± 0.06 ^bc^	55.88 ± 6.59 ^ab^
Z4	325.00 ± 144.53 ^ab^	25.91 ± 11.75 ^cd^	72.00 ± 16.43 ^b^	6.65 ± 1.23 ^abc^	0.09 ± 0.04 ^bc^	59.58 ± 27.35 ^a^
Z5	270.00 ± 32.98 ^bcd^	38.09 ± 10.48 ^bc^	62.00 ± 5.70 ^bc^	8.24 ± 2.39 ^ab^	0.14 ± 0.05 ^b^	53.70 ± 3.93 ^ab^
Z6	219.00 ± 41.44 ^cde^	19.21 ± 3.89 ^d^	59.00 ± 8.94 ^bc^	5.98 ± 1.96 ^bc^	0.09 ± 0.03 ^bc^	46.86 ± 7.70 ^abc^
Z7	287.00 ± 85.48 ^bcd^	41.61 ± 13.92 ^ab^	68.00 ± 16.05 ^b^	8.03 ± 1.77 ^ab^	0.14 ± 0.02 ^b^	46.94 ± 7.92 ^abc^
Z8	358.00 ± 38.01 ^ab^	53.78 ± 14.27 ^a^	95.00 ± 11.73 ^a^	9.09 ± 3.61 ^a^	0.15 ± 0.05 ^b^	44.08 ± 4.04 ^abc^
Z9	388.00 ± 47.25 ^a^	47.86 ± 18.87 ^ab^	97.00 ± 11.51 ^a^	9.00 ± 2.47 ^a^	0.13 ± 0.06 ^bc^	60.08 ± 5.60 ^a^
Z10	300.00 ± 45.69 ^bc^	17.06 ± 4.18 ^d^	75.00 ± 17.68 ^b^	5.07 ± 1.34 ^cd^	0.06 ± 0.01 ^c^	42.94 ± 11.00 ^bc^

**Table 3:** Effects of different treatments on fruit morphological indices. Different lowercase letters are expressed as the same period at the 0.05 level.

Treatments	Transverse Diameter (mm)	Longitudinal Diameter (mm)	Fruit Shape Index	Fruit Hardness (kg/cm^2^)
CK	50.60 ± 4.11 ^c^	50.18 ± 3.12 ^b^	1.00 ± 0.10 ^a^	2.56 ± 0.12 ^a^
Z1	53.90 ± 6.22 ^bc^	51.66 ± 7.47 ^b^	0.96 ± 0.06 ^ab^	2.50 ± 0.17 ^a^
Z2	55.94 ± 4.19 ^bc^	54.96 ± 2.68 ^ab^	0.98 ± 0.05 ^ab^	2.26 ± 0.12 ^ab^
Z3	55.75 ± 2.33 ^bc^	53.75 ± 2.53 ^b^	0.96 ± 0.05 ^ab^	2.50 ± 0.11 ^a^
Z4	58.24 ± 3.50 ^ab^	55.39 ± 3.86 ^ab^	0.95 ± 0.05 ^ab^	2.34 ± 0.25 ^ab^
Z5	57.70 ± 5.56 ^ab^	56.03 ± 3.86 ^ab^	0.97 ± 0.05 ^ab^	2.36 ± 0.07 ^ab^
Z6	57.16 ± 5.74 ^bc^	55.38 ± 5.27 ^ab^	0.97 ± 0.06 ^ab^	2.32 ± 0.07 ^ab^
Z7	54.51 ± 4.98 ^bc^	53.99 ± 4.00 ^ab^	0.99 ± 0.07 ^a^	1.93 ± 0.19 ^c^
Z8	63.60 ± 12.73 ^a^	60.27 ± 12.73 ^a^	0.95 ± 0.06 ^ab^	2.35 ± 0.06 ^ab^
Z9	60.63 ± 8.65 ^ab^	55.60 ± 8.08 ^ab^	0.92 ± 0.09 ^b^	2.33 ± 0.30 ^ab^
Z10	55.25 ± 6.73 ^bc^	54.42 ± 5.42 ^ab^	0.99 ± 0.08 ^ab^	2.12 ± 0.14 ^bc^

**Table 4:** Effects of different treatments on fruit yield and its components. Different lowercase letters are expressed as the same period at the 0.05 level.

Treatments	Average Fruit Weight (g)	Average Fruit Set per Plant	Yield per Plant (kg)	Number of Fruit Clusters
CK	72.24 ± 10.00 ^c^	4.00 ± 1.05 ^b^	0.298 ± 0.076 ^c^	1.90 ± 0.32 ^c^
Z1	87.49 ± 12.62 ^b^	7.90 ± 1.52 ^a^	0.665 ± 0.069 ^b^	2.50 ± 0.53 ^b^
Z2	97.76 ± 12.11 ^ab^	9.80 ± 2.30 ^a^	0.972 ± 0.192 ^a^	2.90 ± 0.57 ^ab^
Z3	104.88 ± 9.98 ^a^	9.90 ± 2.13 ^a^	1.032 ± 0.152 ^a^	3.00 ± 0.00 ^ab^
Z4	106.87 ± 12.41 ^a^	9.60 ± 2.72 ^a^	1.034 ± 0.270 ^a^	3.00 ± 0.47 ^ab^
Z5	105.81 ± 16.69 ^a^	10.20 ± 2.97 ^a^	0.993 ± 0.242 ^a^	2.60 ± 0.70 ^b^
Z6	100.19 ± 13.29 ^ab^	9.20 ± 1.99 ^a^	0.927 ± 0.110 ^ab^	3.00 ± 0.82 ^ab^
Z7	98.90 ± 14.72 ^ab^	9.30 ± 2.95 ^a^	0.929 ± 0.266 ^ab^	2.80 ± 0.63 ^b^
Z8	106.61 ± 19.59 ^a^	10.20 ± 2.94 ^a^	1.022 ± 0.177 ^a^	3.00 ± 0.67 ^ab^
Z9	105.81 ± 14.40 ^a^	9.50 ± 2.12 ^a^	0.999 ± 0.226 ^a^	2.70 ± 0.67 ^b^
Z10	98.98 ± 13.05 ^ab^	9.10 ± 3.03 ^a^	0.852 ± 0.174 ^ab^	3.40 ± 0.52 ^a^

## Data Availability

The original contributions presented in this study are included in the article and Supplementary Materials. Further inquiries can be directed to the corresponding authors.

## Supplementary Materials

The following supporting information can be downloaded at: https://www.mdpi.com/article/10.3390/plants15203092/s1, Table S1: Abbreviations for the components of tomato growth, yield and quality, and fruit quality indicators used in this study.
